# Structural, optical and charge generation properties of chalcostibite and tetrahedrite copper antimony sulfide thin films prepared from metal xanthates†
†Electronic supplementary information (ESI) available: Chemical structures of the used metal xanthates, additional XRD, SEM-EDX and UV-vis data. See DOI: 10.1039/c5ta05777a



**DOI:** 10.1039/c5ta05777a

**Published:** 2015-11-17

**Authors:** Thomas Rath, Andrew J. MacLachlan, Michael D. Brown, Saif A. Haque

**Affiliations:** a Department of Chemistry and Centre for Plastic Electronics , Imperial College London , Imperial College Road , London , SW7 2AZ , UK . Email: t.rath@imperial.ac.uk ; Email: s.a.haque@imperial.ac.uk

## Abstract

Herein, we report on a solution based approach for the preparation of thin films of copper antimony sulfide, an emerging absorber material for third generation solar cells. In this work, copper and antimony xanthates are used as precursor materials for the formation of two different copper antimony sulfide phases: chalcostibite (CuSbS_2_) and tetrahedrite (Cu_12_Sb_4_S_13_). Both phases were thoroughly investigated regarding their structural and optical properties. Moreover, thin films of chalcostibite and tetrahedrite were prepared on mesoporous TiO_2_ layers and photoinduced charge transfer in these metal sulfide/TiO_2_ heterojunctions was studied *via* transient absorption spectroscopy. Photoinduced charge transfer was detected in both the chalcostibite as well as the tetrahedrite sample, which is an essential property in view of applying these materials as light-harvesting agents in semiconductor sensitized solar cells.

## Abstract 2

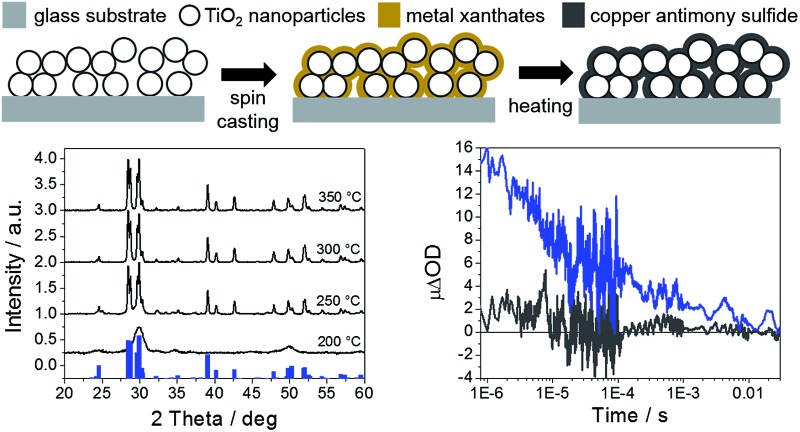
Chalcostibite and tetrahedrite thin films are prepared from solution on mesoporous TiO_2_ layers and photoinduced generation of long-lived charges is detected in these TiO_2_/copper antimony sulfide heterojunctions.

## 1. Introduction

Copper antimony sulfides are interesting absorber materials for sustainable, cost-efficient and scalable photovoltaics as they have high absorption coefficients, well suited band gaps and consist of abundant elements. However, while other metal chalcogenides like cadmium telluride, copper indium gallium sulfide (CIGS) or copper zinc tin sulfide (CZTS) are already well established as solar absorber materials, in contrast, copper antimony sulfide based materials have received limited attention to date. Copper antimony sulfide can be prepared in four different phases, which are CuSbS_2_ (chalcostibite), Cu_12_Sb_4_S_13_ (tetrahedrite), Cu_3_SbS_3_ (skinnerite) and Cu_3_SbS_4_ (famatinite).^1^ These different copper antimony phases have high absorption coefficients over 10^5^ cm^–1^, p-type conductivity and band gaps in the range between 1.1 and 1.8 eV.^1–8^ Among these four phases, the most promising band gap values with respect to highest theoretically achievable power conversion efficiencies (PCEs) are reported for CuSbS_2_ (1.4–1.5 eV)^7,8^ and Cu_12_Sb_4_S_13_ (1.6–1.7 eV).^1,2^

To date, the main focus of research on copper antimony sulfide has been on materials synthesis. Several nanoparticle syntheses have been developed in the last years and the preparation of all four different phases of copper antimony sulfide has been reported.^1–10^ Methods like a hydrazine based route,^7^ sputtering,^11^ thermal evaporation,^12,13^ or sulfurization of electrodeposited metals^14,15^ have already been used for the formation of copper antimony sulfide thin films.

However, to date only a few studies dealt with the characterisation of copper antimony sulfides regarding photoelectrochemistry^2,5,8^ or application of these materials in solar cells.^7,14,16,17^ For CuSbS_2_ based thin film solar cells, a maximum PCE of 3.1% has been achieved so far.^14^ The same PCE was reported very recently for a metal sulfide sensitized solar cell with CuSbS_2_ as absorber material, which highlights the potential of CuSbS_2_ for this type of solar cells.^17^ As such, this presents a strong case for investigating copper antimony sulfides with focus on their application as absorbers in semiconductor sensitized solar cells.

In this work, we report a facile solution based route for the formation of copper antimony sulfide films using metal xanthates as precursors. In particular, we use this approach to fabricate copper antimony sulfide sensitized mesoporous metal oxide films. The presented route is fundamentally different to colloidal nanoparticle syntheses towards copper antimony sulfide, as in this approach, the copper antimony sulfide phases are formed in a solid state reaction directly on the substrate or in the mesoporous metal oxide scaffold without capping ligands. Metal xanthates are versatile metal–organic precursors for the formation of metal sulfides due to their solubility in various solvents^18^ and conversion to metal sulfides at relatively low temperatures (140–200 °C)^19^ and even at room temperature using UV-light treatment.^20^ A variety of metal sulfides have been synthesized employing this method including a range of binary metal sulfides,^21–34^ ternary (CuInS_2_)^35–37^ and quaternary metal sulfides (CZTS).^38^ Moreover, this solution based route employing metal xanthates was initially used to prepare nanocomposites of metal sulfide nanoparticles in organic/polymeric matrices for the application in bulk heterojunction hybrid solar cells,^21,39,40^ but this method is also very well suited to prepare thin metal sulfide films on mesoporous metal oxide scaffolds for application in semiconductor sensitized solar cells.^41,42^

In this paper, we report the synthesis of two copper antimony sulfide phases, chalcostibite and tetrahedrite. We show that by tuning the copper xanthate to antimony xanthate molar ratio two distinct phases of copper antimony sulfide can be realized – namely chalcostibite (CuSbS_2_) and tetrahedrite (Cu_12_Sb_4_S_13_). Furthermore, we present a detailed structural and optical characterisation of these materials by X-ray diffraction, Raman spectroscopy, EDX measurements and UV-vis spectroscopy. Moreover, we also report on transient absorption spectroscopic measurements of photoinduced charge transfer in copper antimony sulfide/mesoporous TiO_2_ films. Evidence of photoinduced charge transfer across the copper antimony sulfide/mesoporous TiO_2_ heterojunction demonstrates the potential of such architectures for solar cell applications.

## 2. Experimental

### 2.1. Materials synthesis

#### Synthesis of copper and antimony xanthates

Copper(i) *O*-2,2-dimethylpentan-3-yl dithiocarbonate was synthesized according to literature.^35^ Synthesis of antimony(iii) *O*-propan-2-yl dithiocarbonate: potassium hydroxide was stirred in a 1 : 1 molar ratio with 2-propanol and a small amount of water. The solution was cooled in an ice bath followed by a dropwise addition of carbon disulfide to a slight excess (1.1 equiv.). The resulting slurry was stirred for 30 min before vacuum filtering and washing with diethyl ether. The potassium *O*-propan-2-yl dithiocarbonate was then recrystallized from methanol. To form the antimony xanthate, an aqueous solution of SbCl_3_ (acidified with HCl conc. to facilitate dissolution of SbCl_3_) was added to a rapidly stirring aqueous solution of the potassium xanthate in a molar ratio of 1 : 3 and stirred for 60 min at room temperature, whereby a precipitate is formed. The pale yellow product was filtered and washed with water followed by methanol. The product was then recrystallized from acetone.

^1^H NMR (400 MHz, CDCl_3_, *δ*): 5.64–5.55 (m, 3H, 3x CH), 1.44 (d, 18H, 6x CH_3_) ppm. Elem. anal. calc. for SbS_6_O_3_C_12_H_21_: C 27.33, H 4.01; found: C 27.38, H 4.09.

#### Preparation of copper antimony sulfide thin films

Precursor solutions were prepared by dissolving copper(i) *O*-2,2-dimethylpentan-3-yl dithiocarbonate and antimony(iii) *O*-propan-2-yl dithiocarbonate separately in chlorobenzene (typical concentrations 0.18 or 0.36 mmol mL^–1^). For the formation of chalcostibite the solutions were mixed in a Cu : Sb molar ratio of 1 : 1 and for the formation of tetrahedrite a molar ratio of 3 : 1 was needed. Subsequently after blending the solutions, 1 vol% of *n*-hexylamine (with regard to the amount of chlorobenzene) was added to obtain a stable solution. Thin films of copper antimony sulfide were formed either on bare glass substrates or glass substrates covered with mesoporous TiO_2_ or mesoporous ZrO_2_ layers. Therefore, the precursor solutions were spin casted or drop coated on the respective substrates and heated on a hot plate to 300 °C for 15 min (200–350 °C for the XRD study) in N_2_ atmosphere in a glove box, whereby the precursors were thermally converted into copper antimony sulfide. By the applied coating techniques, typical layer thicknesses of 50–100 nm (spin coating) and 1–2 μm (drop coating) were obtained.

Mesoporous TiO_2_ or ZrO_2_ layers were prepared by spin casting a TiO_2_ paste (30 NR-D, Dyesol) diluted with terpineol (1 : 2.5 w : w) or a ZrO_2_ paste (Zr-Nanoxide Z/SP, Solaronix) diluted with terpineol (1 : 2.5 w : w) on glass substrates. After spin coating, the films were dried for 5 min at 80 °C on a hot plate before they were sintered at 450 °C for 1 h in a furnace in ambient atmosphere.

### 2.2. Characterisation

^1^H NMR spectra were recorded on a 400 MHz Bruker Avance spectrometer. Elemental analyses were carried out on a Universal CHNS Elemental Analyzer Vario El III. X-ray diffraction (XRD) patterns were measured on a PANalytical X'Pert Pro MRD diffractometer using Ni filtered Cu K_α_ radiation at 40 kV and 40 mA. Raman spectroscopy was performed on a LabRAM Infinity spectrometer (Horiba) using a 633 nm He–Ne laser. Scanning electron microscopy-electron dispersive X-ray (SEM-EDX) measurements were carried out on a JEOL 6400 scanning electron microscope operated at 20 kV. Scanning electron microscopic images were acquired on a LEO 1525 Field Emission Scanning Electron Microscope operated at 5 kV using an In Lens detector. Samples for SEM characterisations were coated with chromium (5 nm) by sputtering before the measurements.

Transmittance and reflectance spectra for the determination of the optical absorption coefficient as well as the absorption spectra were recorded on a Shimadzu 2600 spectrophotometer equipped with an ISR-2600Plus integrating sphere attachment. The optical band gaps were estimated from (*αhv*)^2^ *vs. hv* plots by extrapolating the linear part of the function. The copper antimony sulfide films used for the band gap determination had a thickness of 60 nm. Layer thicknesses were measured using a Veeco Dektak surface profiler.

Microsecond transient absorption spectroscopy (μs-TAS) measurements were performed by exciting the samples in inert atmosphere using a dye laser (Photon Technology International Inc. GL-301) pumped by a nitrogen laser (Photon Technology International Inc. GL-3300). A 100 W quartz halogen lamp (Bentham, IL 1) with a stabilized power supply (Bentham, 605) was used as a probe light source. A silicon photodiode (Hamamatsu Photonics, S1722-01) was used to detect the probe light passing through the sample and the signal was amplified before being passed through electronic band-pass filters (Costronics Electronics). The amplified signal was collected with a digital oscilloscope (Tektronics, DPO3012), which was synchronized with a trigger signal from the pump laser pulse from a photodiode (Thorlabs Inc., DET210).

## 3. Results and discussion

**Fig. 1:**
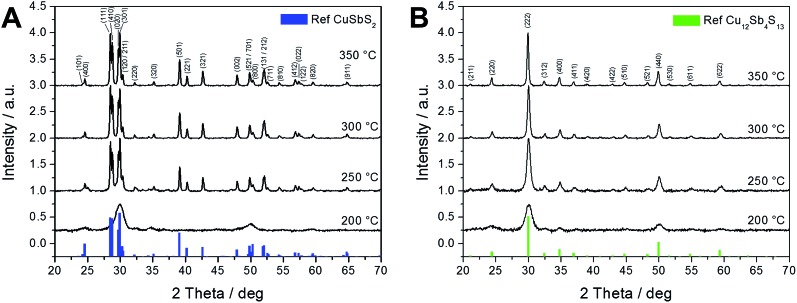
X-ray diffraction patterns of chalcostibite (A) and tetrahedrite (B) layers annealed at different temperatures (200–350 °C) along with the corresponding reference patterns (CuSbS_2_: PDF 00-044-1417; Cu_12_Sb_4_S_13_: PDF 01-088-0282). The main peaks in the patterns are labelled with their Miller indices.

Fig. 1 shows the X-ray diffraction patterns of the prepared copper antimony sulfide phases chalcostibite and tetrahedrite, the phases with the most promising properties for photovoltaic applications. The formation of these two materials could be realized by adjusting the molar ratios of the copper and antimony xanthates (the corresponding chemical structures are given in Fig. S1 in the ESI†). For the preparation of copper antimony sulfide, a copper xanthate bearing a 2,2-dimethylpentyl alkyl side chain and an antimony xanthate carrying an isopropyl alkyl moiety, were chosen. By mixing these two compounds in a chlorobenzene solution, a stable precursor solution for spin casting the precursor films was obtained by adding 1 vol% of *n*-hexylamine, which reacts with the metal xanthates to dialkylthiocarbamates.^19^ These particular compounds were chosen because (i) copper xanthates with shorter, non-branched side chains are not soluble in chlorobenzene,^35^ and (ii) using antimony xanthates with shorter linear side chains, *e.g.* antimony ethyl xanthate leads to precipitation of the copper xanthate in the chlorobenzene solution. Antimony 2,2-dimethylpentyl xanthate would result in a stable precursor solution in combination with copper 2,2-dimethylpentyl xanthate, however, the synthesis of this compound resulted in a liquid oily product and it was not possible to isolate it in crystalline form.

Using a 1 : 1 molar ratio of the selected copper 2,2-dimethylpentyl and antimony isopropyl xanthate leads to chalcostibite (CuSbS_2_) while a 3 : 1 molar ratio results in the formation of the tetrahedrite (Cu_12_Sb_4_S_13_) phase. The formation of these copper antimony sulfides proceeds *via* a solid state reaction whereby the involved metal xanthates in the precursor layer decompose upon thermal treatment and are converted into the respective metal sulfides. The by-products of the thermal decomposition are volatile and leave the layer during the heating step.^21,35^

It is apparent that the X-ray patterns of the samples (molar ratios of 1 : 1 and 3 : 1) heated to 200 °C for 15 min appear quite similar. The peaks are rather broad, which indicates very small crystallite sizes (as estimated using Scherrer equation) and/or not fully developed crystal structures with partly amorphous character at this temperature. However, in the X-ray diffractograms of samples heated to 250 °C, the characteristic peak patterns of the different copper antimony sulfide phases can be clearly identified (Fig. 1A and B). The sample in Fig. 1A, prepared using a molar ratio of copper and antimony xanthate of 1 : 1, exhibits an orthorhombic crystal structure matching well with the reference pattern for chalcostibite (PDF 00-044-1417). The material prepared using a 3 : 1 molar ratio (Fig. 1B) shows a cubic crystal structure of tetrahedrite (PDF 01-088-0282). Moreover, the peaks become much narrower indicating an increased primary crystallite size for the samples annealed at 250 °C. Compared to an alternative solution based method for the fabrication of CuSbS_2_ using metal chlorides and thiourea as precursors,^17^ the metal xanthate based route reported herein has the advantage that crystalline phase-pure material (according to the XRD pattern) is obtained by heating the samples to 250 °C, while for the metal salt based route an annealing step at 500 °C was necessary to avoid the secondary phase Sb_2_S_3_.

It can be seen in Fig. 1A that upon increasing the annealing temperature from 250 °C to 300 °C and 350 °C, the X-ray patterns of the chalcostibite samples show little or no change indicating no further evolution of the crystallite sizes. However, in the tetrahedrite sample, the full width at half maximum (FWHM) of the peaks becomes narrower with higher temperature treatment. Fig. S2† shows the primary crystallite sizes of the samples as a function of annealing temperature. According to an estimation using Scherrer equation (the FWHMs of the 501 and the 222 peaks of chalcostibite and tetrahedrite were used, respectively) the primary crystallite sizes are approx. 70 nm in the chalcostibite samples annealed at 250, 300 and 350 °C, while the sizes increase from 15 to 45 nm in the tetrahedrite sample. This shows that the presented method affords control of the nanocrystal size of tetrahedrite by the applied temperature.

**Fig. 2:**
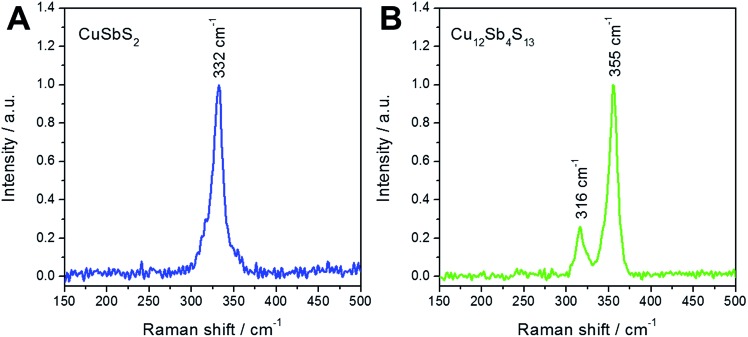
Raman spectra of chalcostibite (A) and tetrahedrite (B) layers annealed at 300 °C.

Fig. 2 shows the Raman spectra of both copper antimony sulfide phases prepared at 300 °C. The chalcostibite sample exhibits a peak at 332 cm^–1^, while in the Raman spectrum of the tetrahedrite sample two peaks at 316 cm^–1^ and 355 cm^–1^ are observed. The Raman spectra presented in Fig. 2A and B are in agreement with reference data.^43^

Next, the chemical compositions of the thin films were analysed *via* SEM-EDX spectroscopy. The EDX spectra (Fig. S3, ESI†) show the characteristic X-ray lines of the three elements copper, antimony and sulfur. In order to determine the relative amounts of copper, antimony and sulfur in the films, Cu K, Sb L and S K series were used. In this way, the chemical compositions of both samples were determined and compared to the theoretical values (see Table 1). As expected, for both chalcostibite and tetrahedrite samples the Cu : Sb : S ratio matches well with theoretical values.

**Table 1:** Chemical composition of CuSbS_2_ and Cu_12_Sb_4_S_13_ determined by SEM-EDX measurements

Material		Cu/at%	Sb/at%	S/at%
CuSbS_2_	Sample	25.7 ± 0.3	25.3 ± 0.2	49.1 ± 0.5
	Theoretical	25.0	25.0	50.0
Cu_12_Sb_4_S_13_	Sample	39.3 ± 0.4	14.1 ± 0.6	46.5 ± 0.4
	Theoretical	41.4	13.8	44.8

**Fig. 3:**
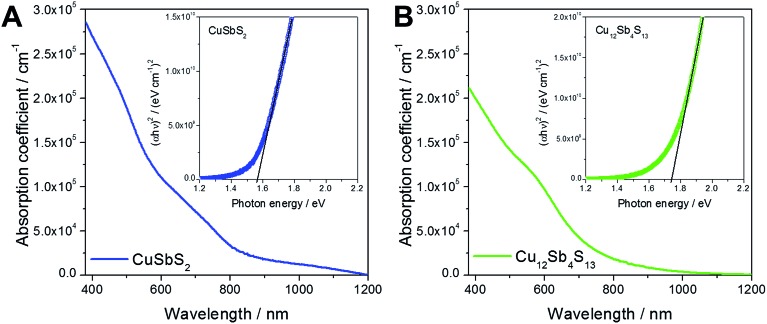
Absorption coefficients of chalcostibite (A) and tetrahedrite (B) thin films annealed at 300 °C and the corresponding Tauc plots for band gap determination shown as insets.

The absorption properties of the chalcostibite and tetrahedrite thin films deposited on glass are presented in Fig. 3. The chalcostibite sample shows a broad absorption over a wide wavelength range with an onset at approx. 800 nm. The band gap values were estimated using Tauc plots, which are shown as insets in Fig. 3A and B. This analysis leads to a band gap of 1.57 eV for the chalcostibite sample. The soft absorption onset at higher wavelengths suggests also an indirect band gap of the material, which is approx. 1.1 eV (see Fig. S4, ESI†). Similar values for direct and indirect band gap are also reported for chalcostibite nanoparticles prepared *via* colloidal synthesis routes.^1,5,9,44,45^ Compared to the chalcostibite sample, the absorption onset of the tetrahedrite sample is slightly blue-shifted and a direct band gap of 1.74 eV was determined as expected for this material according to previous reports on bulk tetrahedrite as well as nanoparticles.^2,46^ No indirect band gap could be determined for the tetrahedrite sample by linear fitting of (*αhν*)^1/2^ as a function of the photon energy which is consistent with the literature.^1^ The absorption coefficients of both copper antimony sulfide samples are higher than 10^5^ cm^–1^ in the wavelength range under 600 nm. These high absorption coefficients together with well-suited band gaps make both phases interesting for PV applications.

**Fig. 4:**
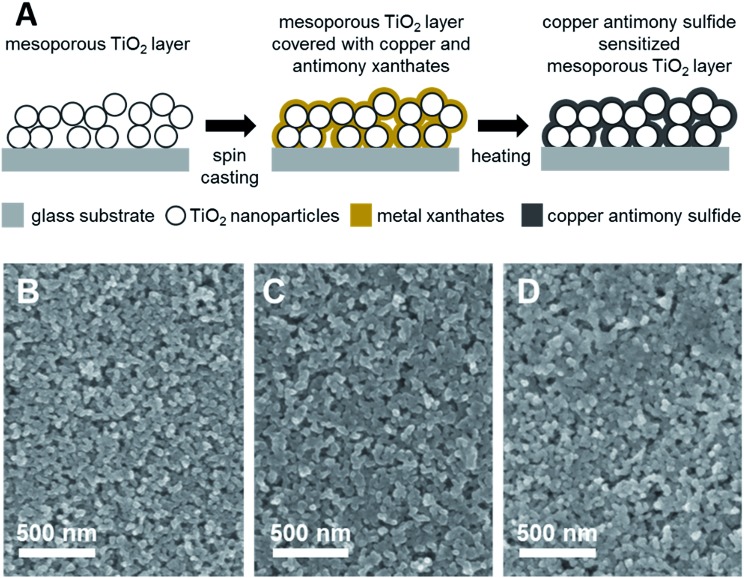
Scheme illustrating the formation of a thin metal sulfide film on a mesoporous metal oxide scaffold using metal xanthates as precursors (A); SEM images of a mesoporous TiO_2_ film (B), covered with a thin film of chalcostibite (C) and tetrahedrite (D). The copper antimony sulfide films were annealed at 300 °C.

We now consider the interfacial charge transfer processes in copper antimony sulfide sensitized TiO_2_ architectures. For this purpose, laser based microsecond–millisecond transient absorption spectroscopy was employed.^47–49^ Thin films of chalcostibite and tetrahedrite were prepared on mesoporous TiO_2_ films as schematically illustrated in Fig. 4A. In a first step, the metal xanthate containing precursor solution was spin casted onto the mesoporous TiO_2_ films whereby the TiO_2_ nanoparticles are covered by the metal xanthates. Subsequent thermal treatment leads to the formation of metal sulfide sensitized TiO_2_ films. SEM images of a bare mesoporous TiO_2_ layer as well as mesoporous TiO_2_ layers sensitized with chalcostibite and tetrahedrite (thicknesses of TiO_2_ films: approx. 1 μm) are shown in Fig. 4B–D. Comparing the SEM image in Fig. 4B with the images in Fig. 4C and D, it can be seen upon careful inspection that the TiO_2_ nanoparticles are covered with a thin film of the metal sulfide. Moreover, it was confirmed by X-ray diffraction measurements that chalcostibite and tetrahedrite are also formed on the mesoporous TiO_2_ layer in the same way as on the glass substrates. The X-ray diffraction patterns of chalcostibite and tetrahedrite prepared on mesoporous TiO_2_ are shown in Fig. S5 in the ESI† and the diffraction peaks are in line with the ones observed for the copper antimony sulfides prepared on glass (Fig. 1), barring the presence of two additional peaks stemming from TiO_2_ at 25.4 and 38.1° 2 theta. Absorption spectra of the chalcostibite and tetrahedrite films prepared on mesoporous TiO_2_ are depicted in Fig. S6.†

**Fig. 5:**
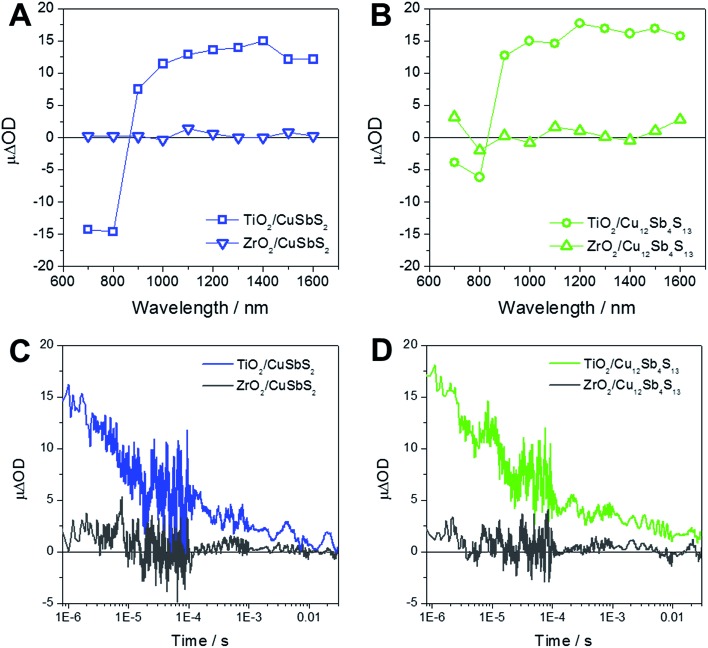
Transient absorption spectra of mesoporous TiO_2_ and ZrO_2_ layers sensitized with CuSbS_2_ (A) and Cu_12_Sb_4_S_13_ (B) measured 1 μs after pulsed laser excitation (wavelength: 450 nm) and the corresponding transient absorption kinetics (C and D) measured using a probe wavelength of 1400 nm.

Fig. 5 shows the results of the transient absorption spectroscopic investigations. Fig. 5A and B show the transient absorption spectra of TiO_2_/chalcostibite, TiO_2_/tetrahedrite, ZrO_2_/chalcostibite and ZrO_2_/tetrahedrite samples measured 1 μs after pulsed laser excitation (wavelength: 450 nm and laser fluency: 15 μJ cm^–2^). The transient absorption spectra of the TiO_2_/copper antimony sulfide heterojunctions show a broad absorption feature between 900 and 1600 nm. This broad absorption feature in this wavelength range is typical for electrons in TiO_2_ or holes in metal sulfides and the observed transient absorption spectrum is comparable to the spectra reported for mesoporous TiO_2_ sensitized with the binary metal chalcogenides CdS, Sb_2_S_3_ or Sb_2_Se_3_.^41,50,51^ The ΔOD values of the ZrO_2_/copper antimony sulfide reference samples are negligible over the whole wavelength range further indicating charge photogeneration in the TiO_2_ based assemblies. The lack of any transient absorption signal in the ZrO_2_ based films is consistent with the higher conduction band energy of ZrO_2_ (relative to TiO_2_)^52^ prohibiting electron injection from copper antimony sulfide to ZrO_2_.

Fig. 5C and D show the charge recombination kinetics (ΔOD, difference in optical density, *versus* time profile) in chalcostibite and tetrahedrite sensitized mesoporous TiO_2_ and ZrO_2_ films. These data were obtained by monitoring the transient absorption signal at 1400 nm following pulsed laser excitation of the samples (wavelength: 450 nm and laser fluency: 15 μJ cm^–2^). As expected, a distinct signal is visible in the samples prepared on mesoporous TiO_2_ indicating the photoinduced generation of long-lived charges across the inorganic absorber/TiO_2_ heterojunction. Our observation of long-lived charge separation (lifetime in range of 1 ms–10 ms) in the TiO_2_ based assemblies is in agreement with previous reports of charge recombination in TiO_2_/metal sulfide films.^41,50^

Moreover, the long-lived charge separation in both TiO_2_/chalcostibite and TiO_2_/tetrahedrite films suggest that chalcostibite and tetrahedrite are well suited as light absorbers in semiconductor sensitized solar cells. As such, the use of these materials is particularly advantageous compared to the well-studied current state of the art metal sulfide based material Sb_2_S_3_,^53,54^ as chalcostibite and tetrahedrite offer the prospect of superior light harvesting due to their smaller band gaps.^17^

## 4. Conclusion

Two phases of copper antimony sulfide, chalcostibite (CuSbS_2_) and tetrahedrite (Cu_12_Sb_4_S_13_), were successfully prepared as thin films on glass and on mesoporous metal oxide layers *via* a solution based precursor route. Copper and antimony xanthates were dissolved in chlorobenzene and after spin casting the solution on the respective substrates, the metal xanthates are converted into copper antimony sulfide upon thermal treatment. Employing a molar ratio of copper and antimony xanthate of 1 : 1 and 3 : 1 in the precursor solution led to the formation of chalcostibite and tetrahedrite phases, respectively, which was confirmed by X-ray diffraction, Raman spectroscopy and SEM-EDX measurements. The characterisation of the optical properties revealed high absorption coefficients for both phases and bandgaps of 1.57 and 1.74 eV are determined for the chalcostibite and tetrahedrite films, respectively. Based on their optical properties, both phases are well suited for photovoltaic applications, which was the reason for further investigation of the prepared materials focusing on charge photogeneration. Mesoporous TiO_2_ films sensitized with chalcostibite and tetrahedrite were studied using microsecond transient absorption spectroscopy and photoinduced charge transfer could be detected in both heterojunctions.
