# Supplementary material for: Structural, optical and charge generation properties of chalcostibite and tetrahedrite copper antimony sulfide thin films prepared from metal xanthates
Source: J Mater Chem A Mater. 2015 Nov 17;3(47):24155–62. doi: 10.1039/c5ta05777a (PMC4786956; doi:10.1039/c5ta05777a)
Supplement: Supplementary file 1 [file TA-003-C5TA05777A-s001.pdf]

Electronic Supplementary Material (ESI) for Journal of Materials Chemistry A.
This journal is © The Royal Society of Chemistry 2015

Supplementary Information

Structural, optical and charge generation properties of Chalcostibite and Tetrahedrite copper antimony sulfide thin films prepared from metal xanthates

Thomas Rath, Andrew J. MacLachlan, Michael D. Brown, Saif A. Haque

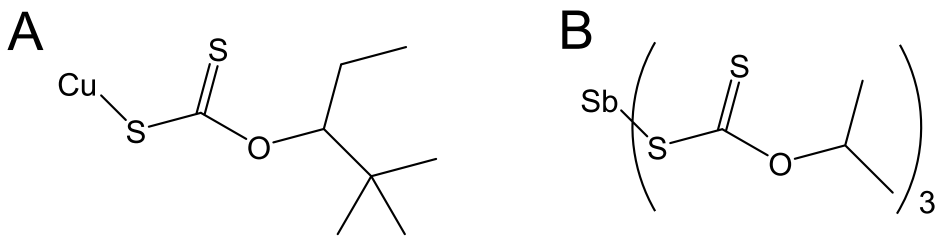

Fig. S1 Chemical structures of the used metal xanthates: (A) copper (I) O-2,2-dimethylpentan-3-yl dithiocarbonate, (B) antimony (III) O-propan-2-yl dithiocarbonate.

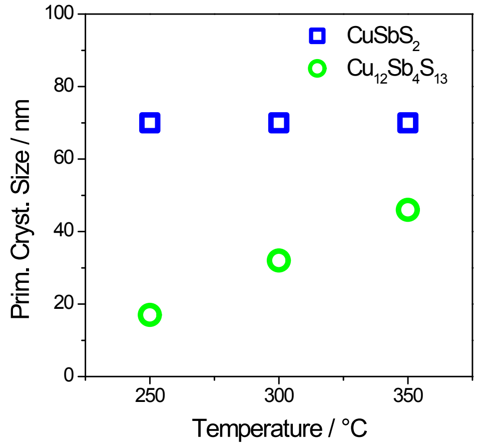

Fig. S2 Primary crystallite sizes of $CuSbS_2$ and $Cu_{12}Sb_4S_{13}$ thin films prepared at different temperatures estimated using Scherrer formula (corresponding XRD patterns are presented in Fig. 1).

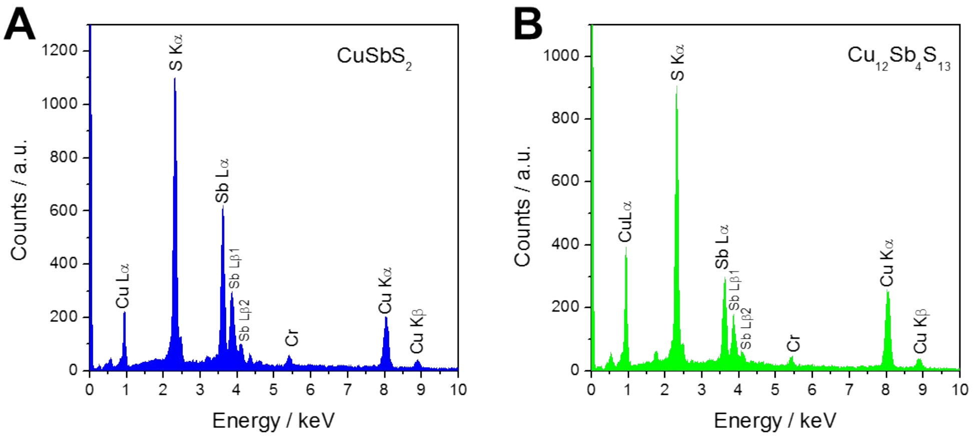

Fig. S3 EDX spectra of Chalcostibite (A) and Tetrahedrite (B) layers annealed at 300 °C. The peaks assigned to chromium stem from a thin film of chromium sputtered on the samples to prevent electrical charging during SEM-EDX characterisation.

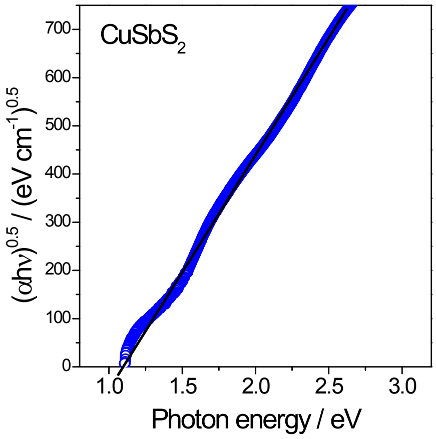

Fig. S4 Tauc plot to determine the indirect band gap of the prepared Chalcostibite film.

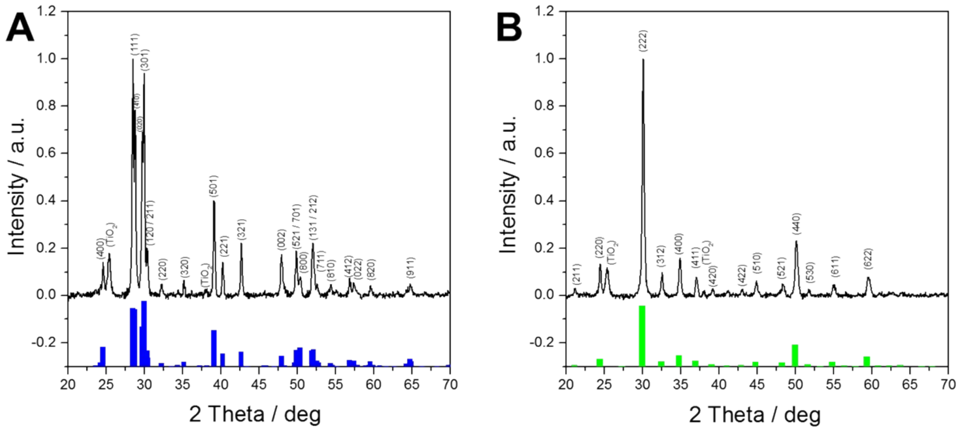

Fig. S5 XRD patterns of $CuSbS_2$ and $Cu_{12}Sb_4S_{13}$ thin films prepared on mesoporous TiO_2 layers. The main peaks in the patterns stemming from $CuSbS_2$ or $Cu_{12}Sb_4S_{13}$, respectively, are labelled are with their Miller indices (reference patterns: $CuSbS_2$ PDF 00-044-1417; $Cu_{12}Sb_4S_{13}$ PDF 01-088-0282). The peaks originating from the mesoporous TiO_2 film are also indicated in the graph.

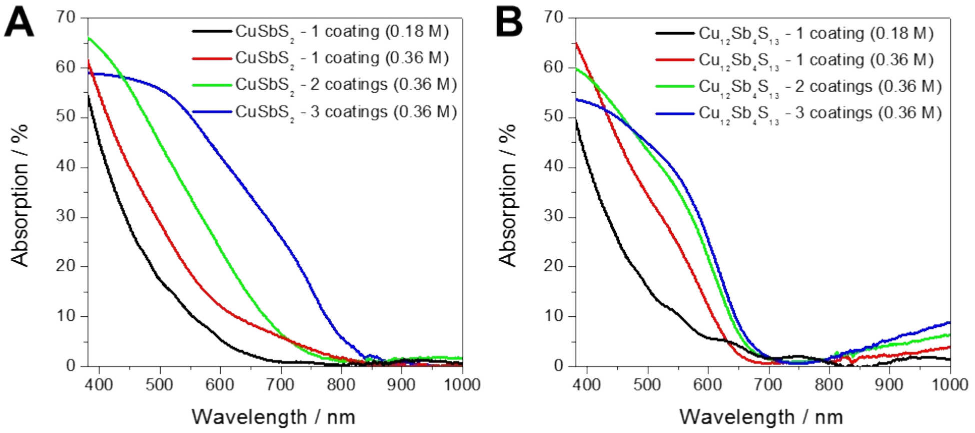

Fig. S6 UV-VIS spectra of $CuSbS_2$ and $Cu_{12}Sb_4S_{13}$ thin films on mesoporous TiO_2 layers. The films were prepared using two different concentrations of the metal xanthates in the precursor solutions (0.18 and 0.36 M) and different numbers of coating and annealing steps. The optical density at 450 nm of the films prepared using a 0.36 M precursor solution is comparable to a 60 nm film of $CuSbS_2$ and $Cu_{12}Sb_4S_{13}$ on a planar glass substrate.
